# Transparent systems, opaque results: a study on automation compliance and task performance

**DOI:** 10.1186/s41235-025-00619-4

**Published:** 2025-02-21

**Authors:** Rebecca L. Pharmer, Christopher D. Wickens, Benjamin A. Clegg

**Affiliations:** 1 Department of Psychology, Colorado State University, 1876 Campus Delivery, Fort Collins, CO 80523‑1876 USA; 2 Department of Psychology, Montana State University, P.O. Box 173440, Bozeman, MT 59717-3440 USA

**Keywords:** Human–automation interaction, Automation transparency, Decision-making, Decision support tools

## Abstract

In two experiments, we examine how features of an imperfect automated decision aid influence compliance with the aid in a simplified, simulated nautical collision avoidance task. Experiment 1 examined the impact of providing transparency in the pre-task instructions regarding which attributes of the task that the aid uses to provide its recommendations. Results showed that transparency here positively influenced compliance with the aid, leading to better task performance. Experiment 2 manipulated transparency via confidence estimates presented alongside the aid’s recommendations. There were no benefits from this form of transparency. In Experiment 2, lower compliance with the aid’s recommendations was found on more difficult collision problems, via a mediating loss of aid reliability and therefore trust. This runs contrary to the hypothesis that harder problems to solve ought to make participants more, rather than less dependent on the aid. Both experiments produced relatively low correlations between trust and compliance. The findings have important implications for the effectiveness of different kinds of transparency implementations, as well as providing a model/framework for understanding how generic factors such as automation reliability and problem difficulty influence both compliance and trust.

## Significance statement

With the increase in prevalence of automated decision support tools, it becomes increasingly important to understand which aspects of these systems influence the human operator to comply or reject their recommendations and the outcome of human compliance on performance. Decision support tools are present in consumer vehicles’ navigation systems, medical diagnostic systems, and—as examined in the present studies—in collision avoidance systems on large vessels. These systems are oftentimes used in high-stakes or safety–critical situations, and failure to calibrate compliance with the true reliability of the system could result in catastrophe (US Navy, 2017). In a maritime collision avoidance scenario, making the wrong decision could result in a loss of life, significant damage, or waste of other resources like fuel. To move the human operator toward better compliance with the aid, we examined how adding automation transparency, or information that explains why a system makes its recommendation, influences not only compliance but also the safety of their maneuvers based on that compliance. From this set of experiments, we found that human operators calibrate their compliance better when they are given a pre-task explanation of the variables the decision support algorithm is considering in its recommendations. However, including a confidence measure that relates to the difficulty of the scenario alongside the system’s recommendations did not lead to further improved compliance or maneuver safety. Findings from this study can inform the design of future systems, and we argue that transparency manipulations need careful consideration to show performance benefits.

## Introduction

The use of automation to assist in decision-making is becoming increasingly prevalent in many facets of life. Whether we are using decision support tools in our vehicle navigation system or in the operating room, it is important to understand the ways in which users interact with these systems. To aid in this understanding, a recent influence model of human–automation team (HAT) performance (Wickens et al., 2024) was developed based on past research. This identified four key features of automation decision aiding systems that influence the human dependence on the automation guidance, and hence, when automation performance is better than that of the unaided human, will influence the performance of the HAT. The four factors are:

1. The reliability of the aid in performing the task requested.
2. The difficulty of the decision or diagnostic problem confronted by automation
3. The transparency of automation, or the degree to which the human operator can understand what automation is doing and why.
4. The degree of automation: whether the aid is merely diagnosing the current state of the process (lower degree) or is also advising what action to take on the basis of this diagnosis (higher degree).

In the current experiments, we examine the first three of these influence factors using a simulated nautical collision avoidance decision aid. We do so in the context of a decision advisor, whereas most of the prior research on these influences has been done only with diagnostic advisors. Below we discuss the first three of these influence factors in detail, before describing the particular collision avoidance task and its decision advisor.

### Reliability of automation

Even high performing automation, or artificial intelligence, if asked to diagnose or suggest solutions to challenging problems will undoubtedly make errors. This is especially likely if automation is designed for prediction, when the future itself is uncertain (e.g., in weather forecasting models, or forecasting the future course of a developing disease). Many recent applications of machine learning algorithms have revealed classification rates well under 100%, when difficult problems are assigned. One general set of conclusions emerging from research on diagnostic automation (Bartlett & McCarley, 2017; Stickland et al., 2021) is that, to the extent that automation reliability increases, humans tend to depend upon automation more. However, they depend upon it less than the higher reliability warrants. Hence, they fall progressively farther short of the level that would be required to achieve an optimal level of dependence, and thus fail to maximize performance of the HAT.

### Difficulty of the task: influence on trust and dependence

A major source of decreasing reliability is the challenge or difficulty of the task that automation is asked to perform. In research on diagnostic aiding, this is often characterized by the measure *d’* from signal detection theory (Bartlett & McCarley, 2017). That is, by an improvement in some combination of the miss rate and false alarm rate of the automation diagnosis, as these measures are frequently employed to evaluate warning systems (Meyer, 2004; Meyer & Lee, 2013; Wickens & Dixon, 2007).

Important to the research questions we ask here, the increase in diagnostic task difficulty can be seen to have two opposing effects (Wickens et al., 2024). On the one hand, it may be that the task is perceived as more difficult by the human member of the team, and this perception will lead them to depend more on the automation. This dependence can occur even to the point of acquiescing to automation when they could discriminate better on their own (i.e., without automation). Wickens and Dixon (2007) refer to this dependency as hanging on to a “concrete life preserver” in the water. On the other hand, more frequent errors made by automation as problem difficulty increases might be evident and salient to the human, as in the case for example of false alerts (Dixon et al., 2007). This may diminish trust in the decision aid, and hence lead to reduced, rather than increased dependence on the automation advice. To the extent that automation performance is nonetheless better than that of the unaided human, this tendency to disregard the advice will degrade performance of the HAT.

It is important to recognize that the extent to which one or the other of these opposing effects occurs depends upon the degree of dissociation between trust and dependency. This dissociation was examined explicitly in a meta-analysis by Patton and Wickens (2024). Two correlational analyses examined experiments that had measured both trust and a performance-based measure of dependency. First, individual differences measures between the two variables showed no significant correlation across the studies. This suggests those people who trusted automation more were not necessarily likely to depend upon it more. Second, examining the covariance across different conditions that experimentally varied in automation reliability, the effect size on trust was over twice that of the effect size on dependence. This implies that there were qualitatively different influences on the two variables in a manner that would be consistent with the two opposing directions of effect described in the previous paragraphs. These differential effects are addressed in Experiment 2.

### Transparency of automation

Automation transparency has been provided various definitions, and we use here that of Lee and See (2004), that transparency communicates information about the automated system to allow operators to form an appropriate mental model and develop appropriate levels of trust. Thorough reviews of the literature, conducted by both van de Merwe et al. (2022) and Bhaskara et al. (2020), have concluded that generally, transparency is helpful in improving the fluency of the HAT. These overall conclusions were buttressed by the results of a meta-analysis carried out on transparency effects by Sargent et al. (2023), both in normal operations and in response to automation failures. They found that introducing or increasing the degree of automation transparency produced a benefit to the accuracy of HAT performance with a large effect size and produced a benefit to the speed of interaction with a medium effect size. Particularly important to the current analysis, transparency significantly increased dependency on automation, as well as increasing the automation user’s situation awareness. This work also identified different forms of transparency such as displaying the “raw data” upon which automation was basing its advice to the human (e.g., Traplsilawati et al., 2017), providing the human with confidence ratings of its own accuracy of judgment (Papadopoulos et al., 2001), or providing instructions on the nature of the algorithms that were deployed by the aid (Meteier et al., 2020). This latter aspect, algorithm instructions, is related to a feature of transparency that we examine here—the simplicity and intuitive nature of the algorithm that guides the aid’s advice. While this feature does not appear to have been explicitly examined in prior transparency research, a plausible case for its effectiveness can be made on the basis of the *binary bias*, introduced and examined by Fisher and Keil (2018). This suggests that people have a cognitive preference for thinking in terms of simple, binary classifications, over more complex continuous ones. In our current context, the simple classification of adhere versus violate the pass behind “rules of the road” for ship navigation, would seemingly be one that is easy to internalize and quite easy to apply in deciding a direction to turn, in contrast to the continuous variables of safety and efficiency in selecting a trajectory to follow. Hence, incorporating this feature into an automation decision aid should create greater transparency. In our two experiments, we address the effectiveness of an aid’s self-confidence assessment, and algorithm transparency (then binary choice) on HAT performance and human dependence.

### Nautical collision avoidance

Ship navigation is a complex task, and for collision avoidance it is particularly so, given the sluggish nature of the ship’s response to steering and speed change commands (Wickens et al., 2019), coupled with the uncertain response of each of the two ships involved in a potential collision. The International Maritime Organization (1977) has employed a set of collision regulations or “rules of the road” to reduce this uncertainty. We model the current paradigm of experiments after two such collision regulations we consider to be most important in the current context: (1) only one ship, that approaching the other from left side, should maneuver to avoid a collision, while the other should maintain constant speed and heading; (2) the maneuvering ship should do so in a manner so as to *pass behind* the other ship. Given the conflict geometry between both ships, this will involve some combination of slowing down, and/or turning right. In implementing these collision regulations, the navigator must also balance two additional factors of *increasing safety*, which means maximizing the distance between the two ships at their point of closest approach (PCA) and *maximizing efficiency,* which means minimizing any changes in speed or heading. Balancing safety and efficiency impose a complex mental calculation (Hockey, et al, 2003; Wickens et al., 2019).

A prior study (Wickens et al., 2020) examined the effectiveness of a decision aid to help advise student “navigators” on the appropriate direction to turn in order to maximize both safety and efficiency. This experiment utilized a simplified collision avoidance task where the user was responsible for avoiding an oncoming ship approaching from the starboard (right) side of the screen. An important feature of any such conflict creating simulation is the difficulty of a given collision problem. Difficulty in conflict avoidance could be objectively measured by the predicted separation of the two ships at their point of closest approach (PCA). If this is large, the choice of maneuver direction will be straightforward. But if it is small, the difference in predicting safety and efficiency between a “turn right” (which will pass behind) and a “turn left” (which will not) will be a difference that is hard to discern. In Wickens et al. (2020), the aid was complied with often, 71% of the time, but less than the 87% reliability of the aid. The safety measure of miss distance at PCA was over twice as high when the aid was complied with than when it was not. The results also revealed that overall participants complied with the “turn right” to pass behind collision regulation (89% of the time) more frequently than when the decision aid recommended violating the heuristics by turning left (63%). This aid rejection, instead favoring the collision regulations, produced a large penalty for safety as the miss distances (MD) were significantly smaller.

In the two experiments to be reported below, based upon Wickens et al. (2020), all participants were initially taught the nature of collision avoidance, and including the two critical collision regulations. In a series of trials, participants then encountered conflict problems in which the optimal maneuver, to maximize safety, was to either turn right to pass behind or turn left to pass in front. On a small percentage of trials, no maneuver was needed at all. The conflict maneuver aid, Caskade, would also recommend a left or right maneuver that would maximize safety and efficiency. Importantly, the aid was imperfectly (87%) reliable, providing the wrong direction on 13% (4 of 32) of the trials in which a maneuver was required, and participants were informed of the imperfection of the aid although not its level of reliability. The conflict problems varied unpredictably in their difficulty, as described by PCA above.

There were three important differences between the two experiments. In Experiment 1, the aid’s decision was informed by the safety and efficiency associated with each maneuver, as in Wickens et al. (2020). However, the Experiment 1 algorithm, unlike in previous versions, now also considered adherence to, or violation of collision regulations in its advice, and participants were explicitly informed of this inclusion. Thus, the upfront instructions incorporated a new level of automation transparency intended to facilitate more calibrated compliance rates than observed by Wickens et al. (2020). This is because maneuver direction was now partly based on a simple, dichotomous, easy to understand dichotomous rule (Fisher & Keil, 2018). Second, in Experiment 2, transparency in the form of automation self-confidence ratings was manipulated, present for half the participants and absent for the other half. Third, while problem difficulty varied unpredictably in both experiments, Experiment 2 explicitly measured this difficulty by the PCA and sorted it into three categories (low, medium, and high) in order to examine which of the two opposing influences of difficulty would dominate: reduced trust because of the salience of automation errors, versus increased dependence because of the automation’s superior abilities with the harder problems. In both experiments, we measured both dependence (compliance with the aid) and subjective measures of trust.

We note here that the methods and results of Experiment 1 were essentially the same as that reported in Pharmer et al. (2021). They are included here: (a) to highlight the replicability of our findings as reported for Experiment 2 and (b) by pooling the results of the two experiments, to signal the robustness of our findings. For Experiment 2, the methods were the same as that reported in Pharmer et al., (2022). However, new analyses not reported there examine the critical effect of task difficulty on trust and dependence.

In the current pair of experiments then, we address four specific hypotheses:

#### H1

(a) Including the procedural variable of collision regulations and explaining its incorporation in more transparent instructions will increase compliance, relative to that observed in Wickens et al., 2020. This comparison is made between the data of the two experiments. (b) Given that Caskade advice is useful, this increase in compliance will improve safety relative to that observed in (Wickens et al., 2020)

#### H2

In Experiment 2, we explicitly manipulate problem difficulty, and we anticipate greater human dependence on automation for the more difficulty problems; even as automation itself performs more poorly on those problems (Barlett & McCarley, 2017; 2021; Boskemper et al., 2021; Munoz Gomez Andrade, et al., 2022).

#### H3

In Experiment 2, we hypothesize that rated trust, dependence, and task performance will all be improved by incorporating the transparency of automation confidence ratings.

#### H1

In both experiments, we correlate, across participants, their self-rated trust with observed behavioral dependence. While we hypothesize a positive correlation, we also anticipate that this will be relatively low, with one variable accounting for less than half of the variance in the other; this is a consequence of the only partial overlap of influences on the two variables.

## Experiment 1

The experimental design detailed in this paper was previously published in shorter form in Pharmer et al. (2021). This study extends upon our earlier work by including analyses based on the manipulations of increased transparency through the use of pre-task instructions and serves as a comparison for Experiment 2.

### Experiment 1 method

#### Participants

Seventy-two undergraduate psychology majors were recruited through a university research participation system to participate for course credit. The study took place online, accessible through the participant’s own computer and lasted approximately 45 min. Figure 2 shows the display participants viewed.

#### Experimental design

Experiment 1 utilized a 2 (direction of recommendation: right or left) X 2 (participant compliance with the automation: comply or not comply) within-subjects factorial design to assess differences in miss distance between procedures. Compliance rates and miss distances were also examined for each type of trial (turn direction recommendation automation correct, automation error, and straight advice).

#### Procedure

The trajectory of the ship updated every 2 s with a movement speed of 0.35 cm/sec. In order to make the trajectory of the two ships more uncertain, we utilized three different conflict geometries: Overtaking (the headings of the ships were similar). Crossing (the oncoming ship was on a course roughly perpendicular to the own ship course). Meeting (the two ships were on generally opposite directional paths, moving toward each other). To make these three different geometries less discriminable, there was some trial-by-trial random variability added to the course of the ships. In addition to this, we used both straight and curved paths to increase uncertainty (Wickens et al., 2019). All these factors were varied in a manner that was unpredictable to the participant.

The algorithm utilized three factors in generating recommendations: miss distance difference, efficiency, and compliance with collision regulations. *Miss Distance Difference* (MDD) is the difference in miss distance between a left turn and a right turn, implemented at a specific time into the maneuver. Caskade would prioritize whichever direction resulted in a larger MD. *Efficiency* minimized delay by reducing the deviation from the original line of heading. *Collision Regulations* gave preference to turning right overturning left. Each of the three attributes could yield a maximum penalty of 1.0 (at their highest level of violation) and a minimum of 0 and was equally weighed across the three factors and then summed. The algorithm compared these summed scores in both turn directions (right or left) and the maneuver yielding the smallest penalty was then recommended to the participant. Participants were instructed on the three factors that would play in their score, but not the scoring algorithm itself.

**Fig. 1:**
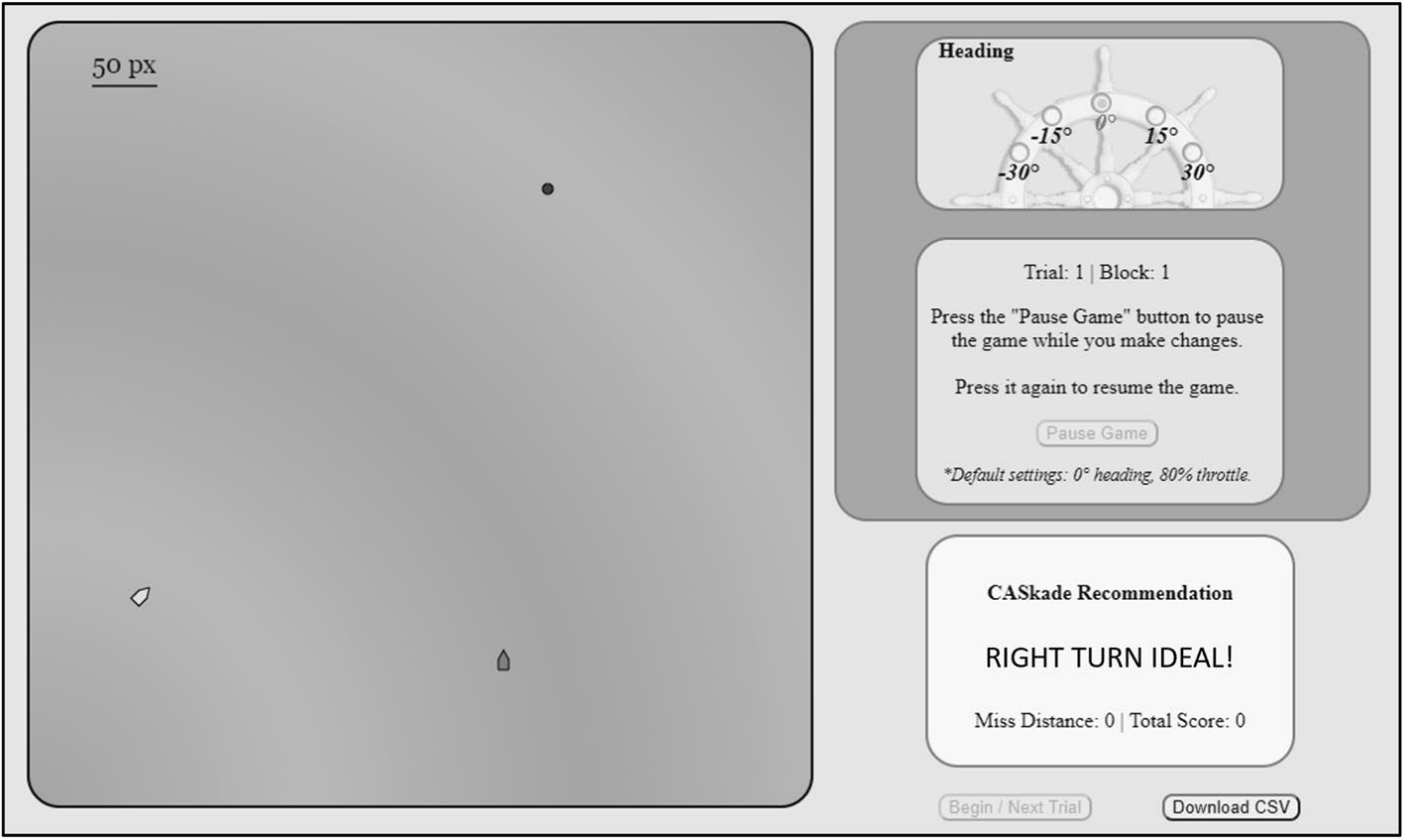
The display of the Caskade testbed. The participant controlled the left ship by using the control panel in the top right corner. The black dot represents the original heading of the participant’s ship. Caskade’s recommendation was provided in the box on the lower right of the screen

The single session included 40 different scenarios presented in a randomized order, 32 of which required the participant to execute a maneuver to avoid a certain collision. Once Caskade made its recommendation at the 16 s mark on each trial, the participant would have 8 s to pause the scenario and execute the maneuver of their choice. As seen in Fig. 1, participants were able to execute both a wide (30 degree) and narrow (15°) turn either to the left or right. Participants were not able to change the speed of their ship. Once the participants had locked in their maneuver by resuming the scenario, they could not change course again. The scenario would resume for another 6 s and participants were presented with feedback on the miss distance, efficiency, and compliance with collision regulations in the form of a score.

Of the 32 trials requiring a maneuver, there were four trials where Caskade would offer incorrect advice (the opposite recommendation of the ideal maneuver, as computed on the basis of safety, efficiency, and collision regulations). This resulted in 28 of the 32 directional trials having correct automated advice, and thus gave Caskade an 87.5% reliability rate for its directional advice. This reliability rate was chosen somewhat arbitrarily, as we aimed for a plausibly reliable system so that participants would not ignore it completely, while keeping the automation imperfect so as to avoid over-reliance on the system. Participants were instructed that Caskade was reliable but not perfect, but not given access to the actual reliability of the system. The remaining eight trials were “straight advice” trials, where Caskade would recommend “Stay on course!”. Participants were instructed that they would still need to pause the scenario to indicate their decision to comply with the straight advice. The straight advice trials were always correct in their recommendation and would never produce a collision, but participants were only instructed to make their best judgment when complying with the advice. If the eight straight trials are included in the reliability equation, reliability could be considered to be 90% (calculated by 36/40 trials).

At the end of each trial, participants were given feedback in the form of a score that aggregated measures of safety (MD), efficiency, and following collision regulations. At the beginning of each subsequent trial, participants would be shown their overall score, comprised of the three aforementioned components. This served as an incentive to increase performance on the three variables as well as a way for participants to gauge the outcome of their compliance or noncompliance with Caskade.

*Automation questionnaire.* To assess individual differences in trust and perceived reliability of the system, we administered a five-item questionnaire at the end of the experimental session. This questionnaire was generated for the purposes of this study and measured perceptions of the specific task and system. Participants responded to the items below on a 10-point Likert scale ranging from “Not at all” to “Always”.

1. Did you trust the automated system to give accurate advice?
2. Did you follow the advice given by the automation?
3. Did you ever feel like the automation was less accurate than 90%?
4. Did you find yourself prioritizing the rules of the road given to you over the advice provided by the automation?
5. Did you find that the automation helped you navigate compared to having no aid at all?

#### Experiment 1 results

The average overall compliance rate on all trials, whether the recommendation was straight or turn, and in the latter case, whether automation was correct or in error was 76%. The average overall safety of performance, as assessed by the miss distance, was 50.7 pixels. Because our interest was specifically in the effects of automation turn direction recommendations, and because straight trials were qualitatively different (no automation errors), we first analyze in detail compliance and safety (miss distance) performance when the automation turn recommendation was correct.

##### Automation correct trials

Table 1 shows, the compliance rates and performance data broken down by the direction of Caskade’s advice, exclusively for the turn recommendations (straight trials are not included). Consistent with the previous study, a paired samples *t* test revealed that overall compliance rates on automation correct trials (72%) are significantly higher on the 28 trials where Caskade correctly recommends a right turn (82%) than trials where Caskade correctly recommends a left turn (61%; *t*(71) = − 27.7, *p* < 0.001, *d* = − 3.27). However, compliance with the left turn recommendations, in violation of collision regulations basic heuristics, has substantially increased to 61%, more than six standard errors higher than the 48% compliance rate reported in the previous version of Caskade (Wickens et al., 2020). That is, when participants are informed that Caskade is taking the collision regulations into account and that the aid weights that factor in its recommendation selection, their compliance rate is now substantially increased when Caskade’s recommendation goes against collision regulations. This shows a more calibrated compliance rate to the actual reliability of the system and more of a reliance on the procedural variable than was observed in the previous study, thereby supporting H1a.

**Table 1:** Compliance rates and resulting miss distances broken down by the direction of Caskade’s advice and the compliance of the user

Caskade advises	Left	Right
Compliance rate (%)	61%	82%
	[SE = 2%]	[SE = 2%]
Miss distance when comply (pixels)	50.9	63.2
	[SE = 1.0]	[SE = 0.4]
Miss distance when not comply (pixels)	42.7	24.3
	[SE = 2.8]	[SE = 1.9]

Miss distance, shown in Table 1, was subjected to a 2 (compliance) X 2 (direction of recommendation) ANOVA. Here, we note that substantial differences in standard error reflect the much smaller N for noncompliance than for compliance. Compliance with Caskade produced a safer (larger) miss distance (*M* = 60.7, *SE* = 0.42) than noncompliance (*M* = 32.1, *SE* = 1.68) (*F*(1,42) = 130.79, *p* < . 05, *η*^*2*^_*p*_ = 0.40). A significant interaction between direction and compliance (*F*(1,42) = 17.5, *p* < 0.05,* η*^*2*^_*p*_ = 0.08) revealed a larger safety penalty (smaller miss distance) when participants rejected a right turn recommendation (*M* = 24.3, *SE* = 1.9), than when they rejected a left turn recommendation (*M* = 42.7, *SE* = 2.8). These miss distances for noncompliance were much greater than those observed in the prior study when collision regulations were not incorporated into the algorithm (17 for left turn, 13.5 for right turn). Thus, better performance, reflecting greater compliance, resulted when the procedural variable was incorporated, thus supporting H1b.

##### Straight advice trials

The average compliance rate across all participants for the eight “straight” advice trials was 74.6%. We performed a paired samples *t* test to assess difference in miss distance between compliance and noncompliance on straight trials, for those participants who both complied and rejected Caskade’s advice at some point in the experiment. Participants’ noncompliance resulted in greater average miss distances (*M* = 68.9, *SE* = 3.62) than compliance (*M* = 32.9, *SE* = 0.72) (*t*(40) = 9.82, *p* < 0.001, *d* = 2.12).

##### Error trials

The compliance rate for the four automation error trials was much lower in the current study (68%), than in Wickens et al., (2020; 85%). The average overall MD on error trials was 50.8% (*SE* = 2.25), not significantly different from the overall average MD on correct trials (*M* = 54.5%, *SE* = 1.32; *t*(61) = 1.34, *p* = 0.18, *d* = 0.25).

##### Individual differences

Using the performance data and participants’ responses to the automation questionnaire, we investigated the relationship between rated trust, compliance, and overall score. The correlation between trust and compliance was *r* = 0.45 (*p* < 0.05), between total score and compliance was *r* = 0.62 (*p* < 0.01), and between trust and score was *r* = 0.36 (*ns*). Of particular interest is the relatively low correlation between trust and compliance. That is, nearly 80% of the variability in compliance is not attributed to variance in trust. This is consistent with our third hypothesis that a substantial dissociation between the two variables exists. The moderate correlation between compliance and total score is to be expected as compliance with the system, which has greater reliability than the unaided human, will generally produce the safest and most efficient maneuvers that also follow procedural collision regulations.

### Experiment 1 discussion

Two primary hypotheses were addressed by the data in Experiment 1: In H1(a), we hypothesized that compliance with automation would improve, relative to that observed in Wickens et al. (2020) in which the procedural variable was not incorporated in the algorithm. This was confirmed, as overall compliance with turn recommendations increased from 66% in Wickens et al (2020), to 72%, an increase specifically attributed to the increased compliance rate when Caskade’s recommendation was in the direction of a left turn, violating the instructed collision regulations. Thus, knowing that the decision aid had “considered” those regulations in formulating its advice and had contradicted them (because of the overriding influence of safety and efficiency on the recommendation for that trial), participants were more compliant with the automation. Such improvement in compliance increased their overall safety of performance (H1b), as gauged by miss distance. This is because of the high reliability of the automation, and, in the correlational analysis, those who complied more had a greater overall score. Hence, we see the advantage of designing decision aiding algorithms to incorporate known, and cognitively simple binary tendencies in the human’s mental model of decision structure and explicitly instructing these prior to the experiment as a form of increasing transparency (Fisher & Keil, 2018). This is, of course, if those tendencies are appropriate.

H4 addressed the correlation between trust and compliance which, while significant and positive, was surprisingly low (*r* = 0.45), an issue we address in the general discussion, to the extent that we replicate this finding in Experiment 2.

Problem difficulty (the predicted miss distance) was varied randomly across trials, and performance was not assessed as a function of this miss distance variable. While participants may have noticed larger differences in predicted miss distances between maneuvers (especially on low-difficulty trials where this distinction is salient), this distinction would be difficult to make without provided online transparency. Hence, our remaining hypotheses (H2 and H3) are examined in Experiment 2.

## Experiment 2

Similar to Experiment 1, this study expands on the short-form version reported in Pharmer et al. (2022), however with significant expansion on the findings as well as further analyses examining automation compliance as a function of problem difficulty that were not previously reported.

Experiment 2 was a replication of Experiment 1; with two important exceptions: changes related to task difficulty (H2) and implementing transparency online (H3). Regarding task difficulty and examining the role of difficulty in influencing automation dependency, we separately coded trials in terms of three levels of difficulty, as operationally defined by the predicted closest point of approach—that is, the miss distance between the ships if the participant exercises no control. The smaller this value, the more difficult it will be to discriminate against the predicted miss distance yielded by a right passage relative to a left passage; and thus determine the safest maneuver (which is, generally, to turn in the direction of that predicted passage). Classifying trial difficulty in this way allows us to examine the effect of automation advice difficulty on user compliance behavior, as seen in previous studies (e.g., Bartlett & McCarley, 2017; Boskemper & McCarley, 2021). Furthermore, because this is highly plausible with imperfect automation in the real world, we have programmed automation error rates to be progressively higher with increase in task difficulty. Such a relationship is ecologically plausible, as whatever logic is analyzing conflict geometry in real time on board a ship will be more likely to err in its trajectory judgment when the difference of maneuver safety is negligible between either passage (ahead or behind). In a real-world scenario, these automation prediction errors would result from such noise factors as turbulent seas, or imperfect location and heading returns from sensors. Thus, on the one hand, the smaller the miss distance difference (MDD) at the predicted point of closest approach, the harder the problem and the *more* likely a human would be to depend on and hence comply with automation, reflecting the automation bias. But on the other hand, such dependence will be based on increasingly *less* reliable automation, a decrease which has been found to cause the human to depend *less* on the advice of automation (Wickens et al., 2024), assuming they notice the automation error. Because such automation errors are programmed to be relatively infrequent, in our analysis, we use the proportion of times the participant complies with these *automation-wrong* trials as an operational measure of the automation bias (Mosier et al., 2017; Parasuraman & Manzey, 2010).

While Experiment 1 established Caskade as a generally effective imperfect decision aid with users complying with its advice an average 72% of the time, users’ compliance was still well below the 87.50% reliability of the system. Our goal is to increase compliance with the aid to be more closely calibrated to the actual reliability of the Caskade algorithm. In addition, optimal performance in a human–automation system requires *synergy* (Boskemper & McCarley, 2021)—where with mostly reliable automation, the *eutactic* human (see Moray, 2003) steps in when clearly needed to override bad recommendations but does not override the good ones. In this case, transparency might serve to highlight conditions in which the decision is more ambiguous and, in doing so, also draw operator attention to confident but wrong recommendations from the automation.

In Experiment 2, to examine H4, we provide half of the participants with numerical ratings of Caskade’s confidence in its own recommendation, a procedure that has shown benefits in some previous research (e.g., Beller et al., 2013; Kunze et al., 2019; Mercado et al., 2016; Papadopoulos et al., 2001; Sarter & Schroeder, 2001; Van der Waa et al.; 2020). To make these measures ecologically plausible, we have inversely correlated such stated confidence values with the difficulty of the conflict trial, as defined by the predicted miss distance above. This allows us to examine H2.

### Experiment 2 method

#### Participants

Fifty-six undergraduate psychology majors were recruited through a university psychology department research participation system to participate for course credit. The study took place online, accessible through the participant’s own computer and lasted approximately 45 min.

#### Procedure

We used a variant of the same maritime collision avoidance task as in Experiment 1. The algorithm utilized the same three factors (miss distance difference, efficiency, and compliance with collision regulations) when providing its recommendation as well as the same scoring logic from the first experiment. The single session again included 40 different scenarios presented in a randomized order, 32 of which required the participant to execute a maneuver to avoid a collision.

**Fig. 2:**
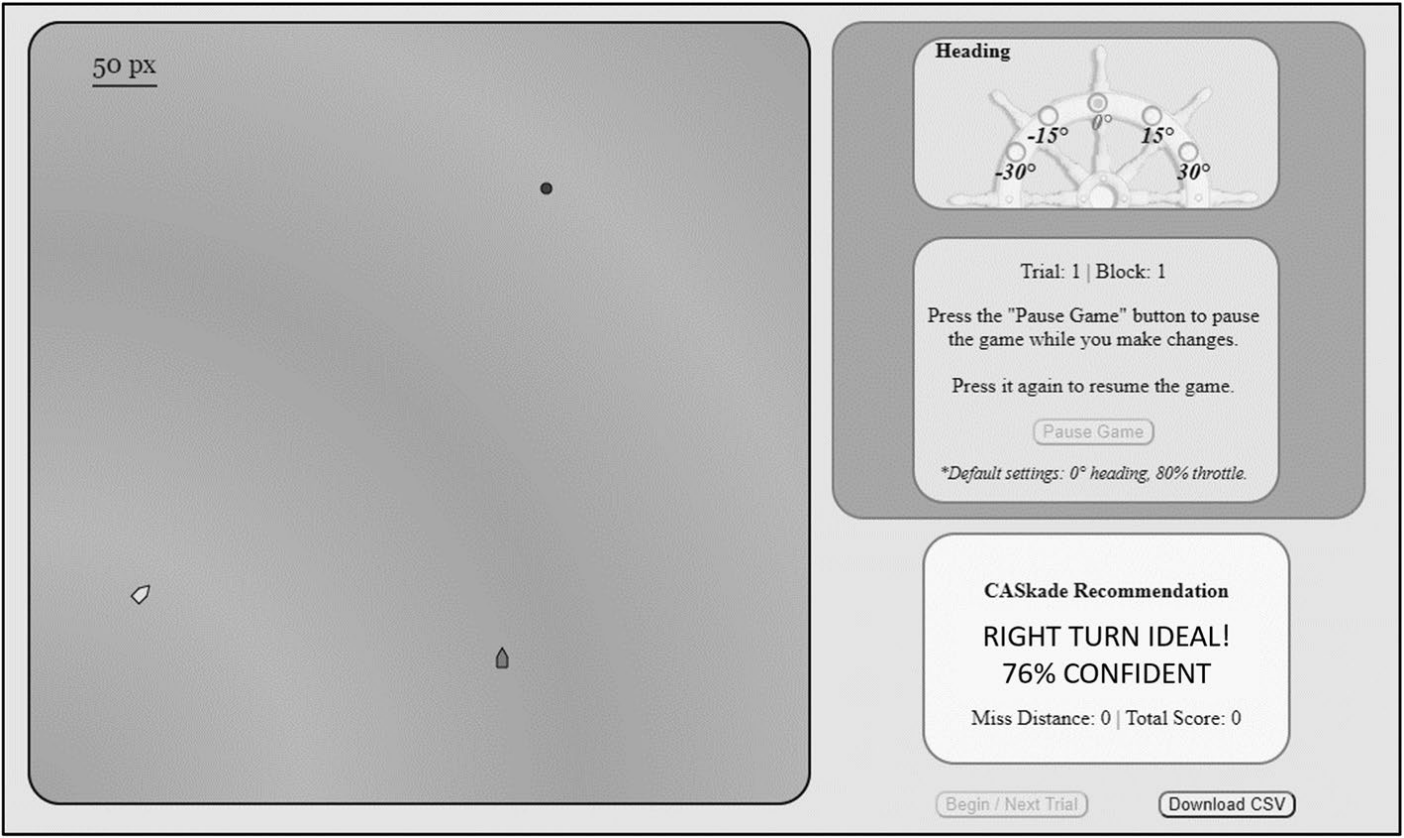
The display of the Maritime Collision Avoidance Task for Experiment 2. The mechanics of the task stayed consistent with those reported in Experiment 1. The key difference being Caskade’s recommendation was now provided along with a confidence measure in the transparency condition. Participants in the control condition viewed the same recommendation without the confidence measure

In the transparency condition, a confidence measure accompanied Caskade’s recommendation on each trial (e.g., Mercado et al., 2016; see Fig. 2). To increase the credibility of this transparency manipulation, the confidence rating was correlated with the actual reliability of the recommendation, and both were correlated with the inferred difficulty of a scenario. In doing this, we note that each scenario in the collision avoidance task produced a different miss distance for either a left or right turn, the *Miss Distance Difference* (MDD). Across all the trials in which automation made a turn recommendation, 24 of 32 or 75% of the recommendations were correct. As in Experiment 1, Caskade never made an error on a “straight” trial (i.e., recommending a turn when a safe passage could be achieved with no correction). Thus, across all 40 trials where Caskade recommends a maneuver, the reliability of Caskade was 80%. Participants were told the confidence value was Caskade’s confidence that its recommendation was the most optimal maneuver in terms of safety, efficiency, and collision regulations. Participants in the control (nontransparency) condition were given similar pre-task instruction that the algorithm was taking these three attributes into account in its recommendations without a mention of confidence.

As described above, scenarios with a small MDD between left and right turns are more difficult for the human to judge and, so, we reasoned, it would also be more challenging for the automation to produce a correct recommendation. That is, to preserve ecological fidelity, automation errors were more likely to occur on more challenging problems, and whatever algorithms were employed, the automated system would be “self-aware” of this degree of challenge. Therefore, these smaller MDD scenarios were accompanied by both a lower automation confidence and a higher automation error rate. For those participants in the transparency condition, 12 trials were classified as difficult, and they provided a wrong recommendation (error rate) 41% percent of the time, and a displayed confidence rating between 65 and 69%. Eleven trials were classified as moderate, having an error rate of 27% and generating a confidence score between 70 and 74%. Nine trials were classified as easy, had a 0% error rate, and assigned a confidence score between 75 and 80%. Participants in the control condition received the same recommendations on each trial as the transparency condition.

##### Experimental design

Experiment 2 utilized a mixed design with a between-subject variable of transparency confidence (present or absent) and a within-subjects variable of direction of Caskade’s recommendation (left or right) to assess differences in the miss distance between procedures. Compliance rates and miss distances were also examined for each type of trial (automation correct, automation error, and straight advice). In addition to the two fixed effects, as in Experiment 1, our analysis also dichotomized between trials in which participants complied, or did not, with the Caskade recommendation, and separately analyzed trials on which automation was correct versus in error. Finally, we examined performance and compliance differences as a function of the three levels of conflict detection difficulty (and hence automation reliability).

##### Automation perceptions questionnaire

At the end of the experimental session, participants were administered the same five-item questionnaire that we created to assess participants’ perceptions of Caskade used in Experiment 1.

### Experiment 2 results

We measured compliance as we had in Experiment 1, by examining the frequency with which users complied with the automation rather than rejecting its recommendations. Across all turn recommendation trials, the compliance rate was 74%, essentially replicating the value of 76% in Experiment 1. Importantly, the compliance rate was not significantly different between the two groups, whether they had transparency (*M* = 75.3%) or not (*M* = 72.2%; *t*(54) = − 0.56, *p* = 0.5, *d* = − 0.49). A Bayesian test revealed credible evidence of no difference between these two levels (*BF* = 0.32).

Table 2 presents the compliance data in the top row, collapsed over transparency conditions, along with the safety performance (miss distance) data when they did and did not comply, as a function of the direction of turn recommendation. The compliance value did differ between directions, showing a strong participant preference for complying with Caskade’s right turn recommendations favoring the collision regulations (*M* = 82.10%, *SE* = 1.12%) over left turn recommendations (*M* = 69.60%, *SE* = 2.29%) (*F*(2, 54) = 52.80, *p* < 0.001, *η*^*2*^ = 0.05). This difference (82–69%) is quite close to the analogous difference observed in Experiment 1 (82–61%).

**Table 2:** Overall compliance rates and miss distances split between Caskade’s recommendation and user action, regardless of condition or trial type

Caskade advises	Left	Right
Overall compliance rate:	69.60%	82.10%
	[SE = 2.29%]	[SE = 1.12%]
Miss distance when complied:	49.30	57.90
	[SE = 0.74]	[SE = 0.42]
Miss distance when rejected:	47.60	61.00
	[SE = 2.60]	[SE = 1.37]

#### Error trials

The previous analysis was conducted over all trials, whether automation was correct or not. To examine performance on the eight total automation error trials where Caskade recommended the suboptimal maneuver, an independent samples *t* test revealed no significant difference in compliance rates between the transparency (*M* = 60.6%, *SE* = 5.5) and the control condition (*M* = 51.3%, *SE* = 6.40; *t*(54) = − 1.10, *p* = 0.27, *d* = − 0.29, *BF* = 0.45). The error trial data, collapsed over transparency conditions, are shown in Table 3.

**Table 3:** Error trials overall compliance rates and miss distances split between Caskade’s recommendation and user action

Caskade advises	Left	Right
Overall compliance rate:	45.90%	53.80%
	[SE = 5.44%]	[SE = 4.91]
Miss distance when complied:	55.90	58.20
	[SE = 1.76]	[SE = 1.10]
Miss distance when rejected:	20.70	20.90
	[SE = 3.22]	[SE = 3.44]

It is notable that the overall compliance rate for automation error trials (*M* = 56.60%) is significantly **less** than compliance on trials when Caskade was correct (*M* = 75.30%;* t*(52) = − 2.34, *p* = 0.02), signaling that the participants were not simply blindly following the aid recommendation, but sometimes became aware of the aid’s error, and rejected its advice. While this difference had not been found to be statistically significant in Experiment 1, it was shown there in the same direction.

#### Correct trials

On the 24 trials where Caskade’s advice was correct, the transparency condition had an average compliance rate of 76.2% (*SE* = 0.01%), which was not significantly different from the average compliance rate of 74.5% (*SE* = 0.01%) in the control condition (*t*(54) = − 0.56, *p* = 0.58, *d* = − 0.15, *BF* = 0.31). There was also no significant difference in the miss distances between the transparency condition (*M* = 50, *SE* = 0.93) and the control condition (*M* = 47.80, *SE* = 0.95; *t*(54) = − 0.73, *p* = 0.47, *d* = − 0.20, *BF* = 0.34). These findings are to be discussed further.

#### Straight advice trials

Eight of the total trials were straight advice trials, in which Caskade did not recommend a maneuver and instead recommended to stay on course. As noted, these recommendations were error free. An independent samples *t* test showed no significant difference between compliance rates in the control condition (*M* = 84.8%, *SE* = 0.02%) and the transparency condition (*M* = 86.4%, *SE* = 0.02%) (*t*(53) = − 0.30, *p* = 0.77, *d* = − 0.08, *BF* = 0.28).

There was also no significant difference in the safety of these trials between the transparency and control conditions. The control condition had an average MD of 40.4 (*SE* = 2.4), and the transparency condition had an average MD of 38.9 (*SE* = 12.5; *t*(53) = 0.56, *p* = 0.58, *d* = 0.15, *BF* = 0.31).

#### Task difficulty effects

**Fig. 3:**
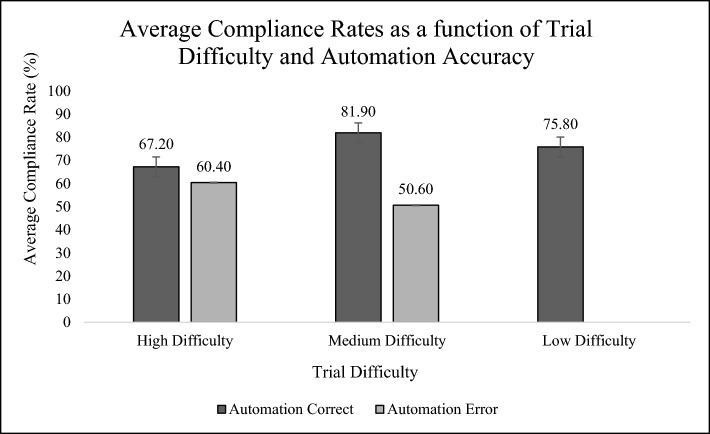
Average Compliance Rates as a function of Trial Difficulty and Automation Accuracy. *Note*: No automation errors were present on low-difficulty trials

Recall that Caskade-expressed confidence was inversely correlated with discrimination task difficulty (MDD). To assess the effects of trial task difficulty on compliance with the aid (see Fig. 3), we conducted a 2 (transparency versus control) × 3 (difficulty: low, medium, high, where higher difficulty indicated lower confidence) ANOVA on compliance rate. To create ecological fidelity, Caskade never made an error on the low-difficulty trials. A main effect of trial difficulty was observed (*F*(2, 108) = 21.36, *p* > 0.001, *η*^*2*^ = 0.09) with the nine lowest difficulty trials showing the highest rate of compliance (*M* = 80.6%, *SE* = 0.03), followed by the 11 medium-difficulty trials (*M* = 73.4%, *SE* = 0.03), and then the 12 high-difficulty trials (*M* = 64.3%, *SE* = 0.04). Planned contrasts revealed that while there was no difference in compliance between low and medium-difficulty trials (for automation correct trials only) (*t*(1) = − 0.64, *p* = 0.52, *r* = − 0.03) there was a significant decrease in compliance from medium to difficult trials (*t*(1) = − 2.6, *p* = 0.01, *r* = − 0.12).

**Fig. 4:**
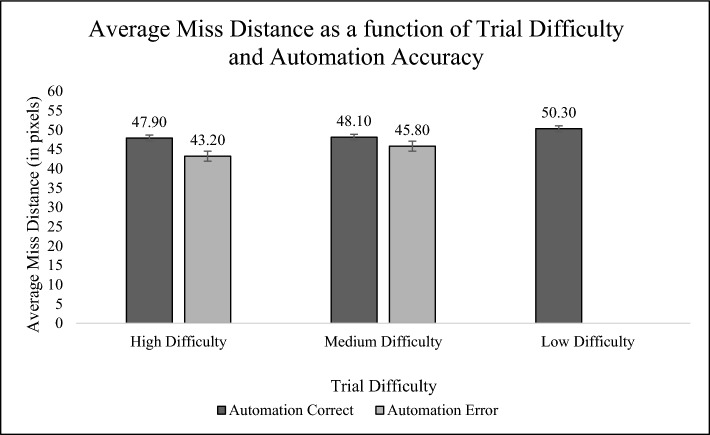
Average Miss Distance (in pixels) as a function of Trial Difficulty and Automation Accuracy. *Note*: No automation errors were present on Low-Difficulty trials

Miss distance data (Fig. 4) revealed a significant main effect of trial difficulty (*F*(1,3) = 32.03, *p* < 0.001, *η*^*2*^ = 0.04), with progressively greater safety for the easier-to-discriminate conflicts. That is, low-difficulty trials had the greatest overall miss distance (M = 50.30, SE = 1.30), followed by medium-difficulty trials (M = 47.40, SE = 0.91), and least safe maneuvers occurring on high-difficulty trials (M = 45.90, SE = 0.94). Whether automation was correct or not had no significant effect on miss distance.

#### Individual differences

We used responses to the automation questionnaire to assess the influence of participants’ rated trust on their compliance with the aid. On the 10-point Likert scale, participants in the transparency condition reported an average trust rating of 7.24 (*SD* = 2.10) while the control condition reported an average trust rating of 8.10 (*SD* = 2.40). An independent samples *t* test found no significant difference in trust ratings between conditions (*t*(40) = 1.23, *p* = 0.23, *d* = 0.38). The average trust ratings found in the current study are close to the average of 7.35 reported in Pharmer et al. (2021), indicating that provision of the transparency confidence measure had no effect on the participant’s trust in the aid.

A significant correlation between trust and behavioral compliance was found in both the transparency condition (*r* = 0.59, *p* < 0.01) and in the control condition (*r* = 0.67, *p* < 0.01), and these values did not differ between transparency conditions. We also assessed correlations between compliance with the aid and average miss distance of participants. In both conditions, we found a significant positive correlation between complying with the aid and the safety of maneuvers (transparency: *r* = 0.59, *p* < 0.001; control: *r* = 0.48, *p* < 0.001; these values did not differ). Thus, in both cases, those who complied more performed more safely, a demonstration of the aid’s utility.

### Experiment 2 discussion

Experiment 2 addressed all four hypotheses presented in the Introduction. Regarding H1, we observed, as we did in Experiment 1 that, compared to the results of Wickens et al (2020), there was greater overall compliance with Caskade when procedural element of the instructed collision regulations was both incorporated into the algorithm and, as an expression of transparency, incorporated into the instructions. This increased compliance was particularly evident for recommendations violating those regulations.

H2, not examined in Experiment 1, was contradicted as originally formulated. Participants did not increase compliance with Caskade on more difficult problems, but in fact, they complied less. We infer that this behavior was triggered by the relative salience of automation error and, perhaps, participants own overconfidence in the capabilities of their own judgments on these more challenging problems. We discuss this further below.

H3 was also disconfirmed by all aspects of the data. Incorporating a transparency display of Caskade’s own confidence (and how this decreased with problem difficulty) influenced neither compliance nor safety, nor did it impact rated trust.

H4 regarding the relationship between trust and dependence was again validated as it had been in Experiment 1. The relationship was positive and significant; but again, relatively weak, indicating that a majority of the variance in dependence could not be accounted for by that in trust.

## General discussion

Across two experiments, we aimed to examine the effects of automation transparency on user compliance and task performance in a maritime collision avoidance scenario. Findings from Wickens et al. (2020) indicated that users showed superior performance on this collision avoidance task when complying with a decision aid called Caskade. However, they showed a bias to follow the set of task procedures they were instructed on prior to the task (turn right to pass behind the oncoming ship) and complied significantly less when the aid suggested to violate these procedures (turn left in front of the oncoming ship).

H1 concerned the incorporation of procedural information into both the algorithm driving the Caskade recommendation and in the payoffs for collision avoidance. Here, transparency was increased by incorporating this information into the instructions. In H1(a), we hypothesized that incorporating in the Caskade algorithm this simple dichotomous procedural variable (generally, turn right to pass behind the conflict stand-on-ship, congruent with the collision regulations) would increase compliance. The dichotomous criterion for which way to steer is easy to understand, and in turn reinforcing the binary bias in human decision-making (Fisher & Keil, 2018). The hypothesis was readily confirmed in both experiments, where overall compliance rates substantially and significantly improved relative to the prior experiment where it was not included. This compliance was particularly improved when Caskade’s advice contradicted the instructed collision regulations (recommending a left turn), given that now participants presumably better understood there would be reasoning behind such a contradiction.

H1b regarding improved performance was not confirmed, with equivalence of miss distance between the current two experiments and the previous one in which the procedural variable was not incorporated. This might be a consequence of the overall accuracy of the imperfect Caskade system being only slightly better than the unaided human (Wickens et al., 2020).

H2, uniquely examined in Experiment 2, addressed the influence of problem difficulty on compliance with the aid and performance on the task. Problem difficulty was implemented by calculating the predicted difference in the distance between ships at the point of closest approach, allowing us to quantify the discriminability between the safest and the least safe maneuver. Our hypothesis was that a more difficult problem would induce greater dependence on (and hence compliance with) automation, based upon findings of between-block differences in detection difficulty observed by McCarley et al., (2017; 2020; Boskamper & McCarley, 2021). In fact, our data revealed the opposite: compliance was reduced on the more challenging collision problems. We interpret this finding as being a result of the mediating role of trust in human choice. Learning, from feedback, that these more difficult trials drove Caskade reliability downward, caused user trust to degrade. Hence compliance degraded as well, as shown in Fig. 3. The reduced compliance on these higher difficulty trials also reduced the safety of performance (Fig. 4). It is interesting to speculate that the reduction, rather than the increase in compliance with greater difficulty, might well represent humans’ own overconfidence in their own ability to cope with problems of greater difficulty, a trend reflected in the collective conclusions of several studies examined by Wickens et al., (2021)

Given the well-known association between reliability, trust, and compliance reported in the literature (Hoff & Bashir, 2015), H3 proposed that user rated trust and dependence on automation, and task performance would be improved by incorporating more transparency into the task. Our findings from Experiment 1 corroborate this hypothesis with regard to algorithm simplicity. Participants were more likely to comply with the system when given offline transparency, indicating that the algorithm included the binary collision regulations variable. However, the additional displayed transparency provided on the per trial basis in Experiment 2 did not produce a further increase in compliance, trust, or performance. This finding stands out as a contradiction to the expectations of adding transparency into a system based on the promising effect size reported in Sargent et al.’s (2023) meta-analysis: medium to large for compliance, small to medium for performance accuracy. While Experiment 2 only examined transparency in the form of confidence measures associated with a decision recommendation, and not other forms of transparency, the literature suggests that providing a simple, easily interpretable transparency manipulation should aid in creating a shared understanding between the user and the automation (Beller et al., 2013; Kunze et al., 2019; Mercado et al., 2016; Papadopoulos et al., 2001; Sarter & Schroeder, 2001; Van der Waa et al., 2020). Although the associated literature offers evidence where transparency is beneficial to automation compliance and task performance, the current findings highlight that transparency is not a one-size-fits-all solution. Indeed, in our analyses of trial difficulties, we found that when users are exposed to changes in system reliability, they are reluctant to calibrate their compliance.

Finally, H4 essentially addressed the relatively low correlation between trust and compliance. Such a low value essentially replicates the conclusion of a meta-analysis conducted by Patton and Wickens (2024) who observed that, across studies, the average correlation was actually far less than that reported here. This is consistent with the Influence model of human–automation team performance proposed by Wickens et al. (2024), which articulates the substantially different influences on trust and compliance. Thus, for example, high workload may increase compliance even as it does not influence trust; and lower reliability may degrade trust, even as it does not degrade compliance.

## Limitations and future directions

This study examined transparency by providing pre-task explanations of the variables used in the algorithm’s decisions and during-task confidence ratings associated with its recommendations. However, alternative approaches to transparency might better support compliance calibration and task performance. For instance, predictive visualizations of maneuver outcomes or simple comparisons of miss distances for different maneuvers could potentially enhance interpretability and might help participants better understand and respond based on difficult scenarios. Future research should investigate whether these or other forms of transparency help users develop a more accurate understanding of the system’s reliability and make safer, more optimal decisions. Additionally, the simplicity of the current task—where the operator only needed to avoid a single ship—may have influenced findings. Expanding the complexity of tasks to include more realistic maritime scenarios, such as navigating crowded waterways with varying numbers of vessels per trial, could yield different results. Another limitation of the study was the use of a novice university student sample, which may not generalize to expert operators in real-world maritime navigation tasks. Experts, with their prior knowledge and experience, might interact with the system differently, potentially leveraging transparency features in unique ways. Future research should examine how expert users interact with this paradigm, as their decision-making strategies and reliance on algorithmic support may differ significantly from those of novices.

## Conclusions

In conclusion, our results revealed that automation transparency exerted mixed success. On the one hand, when incorporated into instructions regarding simple features of the automation algorithm, it appears to substantially benefit the performance of the human–automation team. But on the other hand, when expressed on a trial-by-trial basis as a valid confidence measure reflecting problem difficulty, its incorporation was not effective. However, increasing trial difficulty did adversely affect compliance rate, in a manner suggesting that people are more sensitive to the increasing automation error rate, than they are in their own challenges in performing the task unaided. Finally, we conclude that the current data strongly replicate the general findings of only a modest, albeit significant correlation between trust, and the behavioral measure of compliance.

## Data Availability

The datasets used and/or analyzed during the current study are available from the corresponding author on reasonable request. Please email Rebecca.pharmer@colostate.edu for access to materials.
